# Long-Term Effect of Half-Fluence Photodynamic Therapy on Fundus Autofluorescence in Acute Central Serous Chorioretinopathy

**DOI:** 10.1155/2020/8491712

**Published:** 2020-02-17

**Authors:** Martin Stattin, Stefan Hagen, Daniel Ahmed, Eva Smretschnig, Florian Frommlet, Katharina Krepler, Siamak Ansari-Shahrezaei

**Affiliations:** ^1^Karl Landsteiner Institute of Retinal Research and Imaging, Vienna 1030, Juchgasse 25, Austria; ^2^Department of Ophthalmology, Rudolf Foundation Hospital, Vienna 1030, Juchgasse 25, Austria; ^3^Center for Medical Statistics, Informatics, and Intelligent Systems, Medical University of Vienna, Vienna 1090, Spitalgasse 23, Austria; ^4^Department of Ophthalmology, Medical University of Graz, Graz 8036, Auenbruggerplatz 4, Austria

## Abstract

**Purpose:**

To evaluate normalized short-wavelength fundus autofluorescence (SW-FAF) imaging changes over time as a predictive parameter for the retinal pigment epithelium (RPE) function in eyes compromised by acute central serous chorioretinopathy (CSCR) after indocyanine green angiography-guided verteporfin (Visudyne®, Novartis Pharma, Basel, Switzerland) photodynamic therapy (PDT) with a half-fluence rate (25 J/cm^2^).

**Methods:**

Quantitative data of SW-FAF grey values (SW-FAF GV) from a 350 *μ*m (SW-350) and 1200 *μ*m (SW-350) and 1200 *t*-test was calculated to explore the differences of SW-350 and SW-1200 between one month and the long-term follow-up.

**Results:**

Mean differences (95% CI) in SW-FAF GV between 1 month and 7 years after half-fluence PDT were 0.07 ± 0.11 for SW-350 ([95% CI: −0.002; 0.14], *p*=0.06) and 0.11 ± 0.15 for SW-1200 ([95% CI: 0.01; 0.21], *p*=0.06) and 0.11 ± 0.15 for SW-1200 ([95% CI: 0.01; 0.21], *p*=0.06) and 0.11 ± 0.15 for SW-1200 ([95% CI: 0.01; 0.21], *p*=0.06) and 0.11 ± 0.15 for SW-1200 ([95% CI: 0.01; 0.21],

**Conclusion:**

After 7 years, normalized SW-FAF GV were significantly lower in eyes with resolved acute CSCR treated with reduced-fluence PDT compared to the follow-up after 1 month without correlation to explicit pattern changes or structural damages. Half-fluence PDT remains a safe and considerable treatment option in acute CSCR.

## 1. Introduction

Acute central serous chorioretinopathy (CSCR) is a disease that primarily affects the choroidal blood circulation and is characterized by an elevation of the central neurosensory retina due to subretinal or subretinal pigment epithelium fluid (SRF; sub-RPE) accumulation leading to various visual symptoms and a reduced vision-related quality of life [1, 2]. Although acute CSCR shows a favorable natural regression in the majority of affected eyes, recurrence occurs in approximately 50 percent and can lead to an irreversible RPE damage [3].

Different treatment modalities aim for the underlying pathophysiological mechanism of vessel dilatation with extravasation of fluid which causes choroidal hyperpermeability and try to conquer its related complications in chronicity such as RPE atrophy, choriocapillary defects, and secondary choroidal neovascularization (CNV) [4–7]. Our study group previously elaborated promising results for the treatment of acute symptomatic CSCR with a reduced-fluence rate (25 J/cm^2^) photodynamic therapy (PDT) with verteporfin (Visudyne®, Novartis Pharma AG, Switzerland) in terms of best-corrected visual acuity (BCVA), central subfoveal thickness (CST), contrast sensitivity, and long-term vision-related quality of life [8, 9]. Adverse events like choroidal ischemia, RPE atrophy, or the development of a secondary CNV have been largely avoided using this adopted PDT regimen according to Lim et al. [10].

Fundus autofluorescence (FAF) imaging is a noninvasive imaging technique which enables the illustration of fluorophores—mainly lipofuscin—in the outer retina by employing stimulated light emission [11]. Short-wavelength (SW) FAF is an accepted parameter to adequately evaluate the function of photoreceptors or RPE cells in vivo [12, 13]. Considered to be a surrogate for the metabolic cell status, it can be measured quantitatively as grey scale values (SW-FAF GV) and correlated qualitatively with funduscopic changes and OCT findings as well as various functional parameters [14–16]. Several studies on short-term results described SW-FAF as a more comprehensive indicator for the course of the disease as well as treatment outcomes than BCVA or CST alone [17, 18]. Longitudinal studies are mandatory to objectify the outcome of reduced-fluence PDT over time.

In the light of the above, this study evaluated the long-term treatment effect of indocyanine green angiography- (ICGA-) guided half-fluence PDT for acute CSCR on SW-FAF GV as a representative of the RPE function after 7 years.

## 2. Materials and Methods

### 2.1. Study Design and Patient Selection

**Figure 1:**
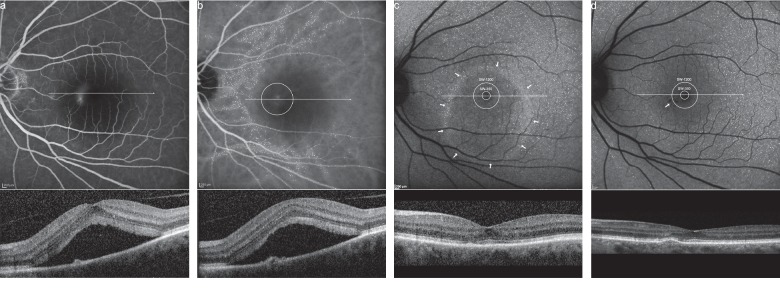
Diagnostic and therapeutic protocol of a left eye with acute CSCR. (a) Late fluorescence angiography with juxta foveal late leakage and a smokestack sign. Below, a spectral domain optical coherence tomography (SD-OCT) slab with subfoveal fluid accumulation corresponding to the bar in the en face image. (b) Midphase indocyanine green angiography showing a 1500 *μ*m laser spot centered at the hyperpermeability. SD-OCT segment through the treatment area with flat pigment epithelial detachment (PED) (arrow). (c) Fundus autofluorescence (FAF) of the same lesion 1 month after treatment with a ring of hyperfluorescence (arrows) demarcating the borders of subretinal fluid extension and two measurement rings encircling the fovea in a 350 *μ*m (SW-350) and 1200 *μ*m (SW-1200) diameter. The short-wavelength (SW) FAF grey values (GV) of both encircled areas were normalized by dividing them through the SW-FAF GV of the complete picture. Resolution of subretinal fluid is visible on the SD-OCT B-scan. (d) The same eye 77 months after treatment with both measurement rings encircling homogenous FAF demonstrating minor hypofluorescent remnants nasal to the fovea (arrow) corresponding to a hyperreflective PED in SD-OCT.

Fifteen Caucasian patients with unilateral acute CSCR were diagnosed and documented by multimodal imaging including SW-FAF, fluorescein angiography (FA), ICGA, and spectral domain-optical coherence tomography (SD-OCT) using a scanning laser ophthalmoscope (HRA + OCT Spectralis, Heidelberg Engineering GmbH, Heidelberg, Germany) at our tertiary eye care center (Medical Retina Unit, Department of Ophthalmology, Rudolf Foundation Hospital Vienna, Austria) between August 2008 and November 2009 [18]. Fifteen eyes were treated for acute symptomatic CSCR involving the fovea by ICGA-guided PDT within 3 months after the onset of symptoms. The location of the lesion was identified by FA (Figure 1(a)), and its size was determined by the existence of choroidal hyperpermeability in ICGA (Figure 1(b)) [5].

The treatment for the area of choroidal hyperpermeability—presumed to be the source of subfoveal fluid—was executed by a modified PDT protocol. A laser light at 689 nm wavelength was applied for 83 seconds with a reduced fluence rate of 25 J/cm^2^ and an intensity of 300 mW/cm^2^. All other parameters including verteporfin at a dosage of 6 mg/m^2^, the infusion time (10 minutes), and the time of laser application (15 minutes after initial drug delivery) followed the standard PDT protocol. All treatments were performed by the same medical retina specialist.

Thirteen patients completed the 1-year visit and were reinvited for a long-term follow-up in 2016. Twenty eyes of 11 patients could be investigated 7 years on average after initial presentation without signs of relapse. No adverse events regarding the development of secondary complications related to the disease or the treatment were documented at the last visit. Informed consent for study inclusion was obtained by all participants, and the study was performed in accordance with the Declaration of Helsinki. All patients underwent a complete ophthalmic examination including best-corrected visual acuity (BCVA) using the Early Treatment Diabetic Retinopathy Study (ETDRS, 4m) —counting every correctly read letter—as well as indirect slit-lamp biomicroscopy with dilated pupils using 0.5% tropicamide (Mydriaticum®, Agepha Pharmaceuticals, Vienna, Austria) and 2.5% phenylephrine drops.

SW-FAF GV and SD-OCT were assessed before half-fluence PDT and again 1, 3, 6, 9, 12, and 83 months after treatment in all but two patients whose data (SW-FAF GV of the contralateral eye) were partially missing. The one-year data have already been published [18]. Patients with CSCR caused by iatrogenic corticosteroids, other macular abnormalities leading to SRF (i.e., neovascular maculopathy and polypoidal choroidal vasculopathy), or patients with a history of ocular surgery were excluded. Bilateral involvement was not applicable for enrollment and initially excluded.

### 2.2. Fundus Autofluorescence

A single operator conducted SW-FAF GV continuously throughout the first year every 3 months and 83 months on average after treatment. Room lights were dimmed to reduce possible side effects and minimize distraction of the tested subject. In the beginning, the fundus was aligned and the foveal depression focused with near-infrared light (820 nm). The laser was then switched to the blue excitation mode, and the image acquired until the whole area reached its maximum intensity. Images were recorded with a 30° view mode and an image resolution of 768 × 768 pixels. An excitation wavelength of 488 nm was used representing blue-peak SW-FAF images, and emitted light with a wavelength above 500 nm was detected with a barrier filter. In each patient, the automated real time mode of the angiograph was activated, and 9 images with a speed of 4.7 frames/s were averaged to obtain high-contrast SW-FAF images.

At each follow-up, mean SW-FAF GV were automatically calculated for two circles of 350 *μ*m (SW-350) and 1200 *μ*m (SW-1200), respectively, in diameter centered at the fovea as well as for the whole SW-FAF image using the built-in software viewer program (Figures 1(c) and 1(d)) of the treated and the untreated eye. Each placement of circles was performed by the same medical retina specialist.

Quantitative SW-FAF GV represented the grey values relative to the maximum possible value and were hence given as percentages of the total. A value of 0 equaled black, and a value of 255 equaled white in the used 8 bit grey value representation. The level of image brightness directly affected SW-FAF GV measurements and varied between visits and patients. Therefore, the mean SW-FAF GV of each circle was normalized by dividing it by the mean SW-FAF GV of the whole image to ensure comparability between visits as described in another study [19]. Hence, a ratio analysis between the foveal (SW-350), respectively, the parafoveal (SW-1200) FAF and the 30° image was performed at every follow-up and compared to each other. Furthermore, this method reduced the bias of variable light absorption due to different opacity in the optical media like cataract formation or corneal haze which could have developed over time in the individual subject. Quantitative SW-FAF is not absolute as it measures not only lipofuscin in the RPE but is theoretically influenced by pathologies of the overlying media including the neurosensory retina.

### 2.3. Spectral Domain-Optical Coherence Tomography

OCT B-scans were routinely conducted using the integrated Spectralis standard SD-OCT device. It utilizes an 880 nm superluminescence diode to simultaneously generate multiple B-scans with a scanning rate of 40.000 A-scans/sec, an axial resolution of 7 *μ*m (optical) and 3.9 *μ*m (digital), respectively, and a transversal resolution of 6 *μ*m (digital). The eye-tracking dual-beam technology (TruTrack™ Active Eye Tracking software) correlates between the OCT scan and the two-dimensional fundus image in a 30° view mode while extracting motion artifacts due to eye movement. The colocalization of posterior structures was immensely helpful to get an impression of the neuroretinal integrity in the first place and for the later monitoring of possible treatment effects.

### 2.4. Statistical Analysis

A 2-sided *t*-test was calculated to analyze the differences of SW-350 and SW-1200 between one month and the long-term follow-up. The analysis of SW-FAF GV before treatment was not carried out in order to avoid a systematic flaw in baseline 30° images due to a hyperautofluorescence related to SRF accumulation. An uncorrected significance level of *α* = 0.05 for both tests was used in order to refrain from correction for multiple testing due to the strong positive correlation between the two time points. Statistical analyses as well as the individual course of SW-350 and SW-1200 at one month and at 83 months were conducted and drawn with R V3.3.2.

## 3. Results

SW-FAF images of 20 eyes in 11 patients were obtained at a median time of 83 (min. 77–max. 89) months after the reduced half-fluence PDT. Baseline FA and ICGA demonstrated extravascular leakage and dilated choroidal vasculature with choroidal hyperpermeability at the macular consistent with acute symptomatic CSCR in 11 eyes (Figures 1(a) and 1(b)). A complete resolution of SRF could be detected in all treated eyes 1 month after PDT. All relevant descriptive data are listed in Table 1.

BCVA before treatment was 0.97 ± 0.24 ETDRS letters and raised to 1.18 ± 0.13 ETDRS letters on average at the last visit. The mean laser spot size for PDT treatment was 1873 ± 403 *μ*m (min. 1500–max. 2600 *μ*m), and the PDT laser spot was placed juxta foveal in all eyes (Figure 1(b)). PDT was well tolerated by all patients without systemic or local adverse events during the verteporfin infusion or the follow-up. No patient developed secondary retinal damages in terms of RPE atrophy, RPE tear, or CNV formation associated with PDT treatment.

**Figure 2:**
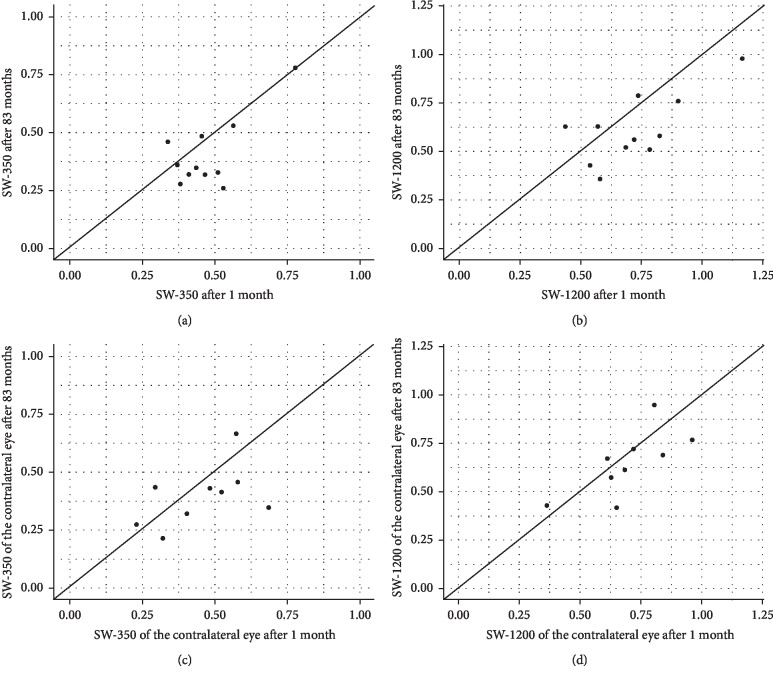
Distribution of normalized short-wavelength fundus autofluorescence grey values (SW-FAF GV). (a) Differences of SW-FAF GV for a 350 *μ*m (SW-350) diameter circle centered at the fovea 1 month vs. 83 months after half-fluence photodynamic therapy (PDT). (b) Differences of SW-FAF GV for a 1200 *μ*m (SW-1200) diameter circle centered at the fovea 1 month vs. 83 months after half-fluence PDT. (c) Differences of SW-350 of the contralateral eye 1 month vs. 83 months. (d) Differences of SW-1200 of the contralateral eye 1 month vs. 83 months.

Mean distribution of SW-350 and SW-1200 was 0.48 ± 0.12 and 0.72 ± 0.2, respectively, 1 month after half-fluence PDT. SW-350 declined to 0.41 ± 0.15 and SW-1200 to 0.61 ± 0.18 on average at the last follow-up 7 years after half-fluence PDT. A significant decrease of normalized SW-FAF GV was observed between the 1-month follow-up and the long-term follow-up in 8 of 11 eyes. Mean differences of SW-350 were 0.07 ± 0.11 (Figure 2(a) [95% CI: −0.002; 0.14], *p*=0.06) and of SW-1200 were 0.11 ± 0.15 (Figure 2(b) [95% CI: 0.01; 0.21], *p*=0.03), respectively.

No influence of age, gender, or spot size could be detected. In 9 contralateral eyes, mean SW-350 measured 0.46 ± 0.15 initially and 0.39 ± 0.13 after 83 months. Mean SW-1200 of the contralateral eye declined from 0.70 ± 0.17 to 0.65 ± 0.17 in the long term. Differences of SW-350 of the contralateral eye were 0.06 ± 0.14 (Figure 2(c) [95% CI: −0.04; 0.17], *p*=0.22) and of SW-1200 were 0.05 ± 0.13 (Figure 2(d) [95% CI: −0.04; 0.15], *p*=0.22), respectively.

## 4. Discussion

In the present work, we reinvestigated the effect of half-fluence PDT in acute CSCR 7 years after initial therapy [18]. BCVA remained stable over time. Normalized SW-FAF GV were used as a marker for the metabolic activity of the RPE. Possible structural RPE changes were explored by SD-OCT on the long run. In contrast to our findings 1 year after therapy, the SW-FAF GV changed significantly and reduced values were found in 8 out of 11 eyes, independently of the measured area (Figures 2(a) and 2(b)) [18]. Normalized SW-FAF GV declined at a smaller (0.07 in SW-350) and even more so at a larger (0.11 in SW-1200) diameter through the umbo of the fovea. As this was a ratio analysis, the longitudinal reduction of SW-FAF GV could be related either to a hypoautofluoresence in the foveal region or a hyperautofluorescence of the total 30° area. No normal range for absolute quantitative FAF exists to date although attempts for uniformity were made. The current equipment and technical changes as well as the repeatability in subjects were only feasible in small sample sizes and not implementable widely in clinical routine. Nevertheless, Delori et al. measured FAF quantitatively in a wide range of subjects and found a significant increase with age [20]. We were not able to distinguish any FAF pattern related to the disease or the treatment, especially in the foveal or parafoveal region. It is known that the correlation for the foveal FAF is less significant when corrected for the optical media as it is variably attenuated by macular pigment absorption. No structural changes related to focal or geographic atrophy could be identified by means of SD-OCT, nor could the laser spot size or its location be matched with SW-FAF GV differences. Zola and her colleagues investigated the evolution of FAF patterns in chronic CSCR over 3 years [21]. They found hypoautofluorescence of two distinct patterns (diffuse or granular) in more than 50% of eyes after 36 months. While hyperautofluorescence was an early finding after 4 months, all other changes including hypoautofluorescence were considered as slow changes. In conclusion to our results, they could not correlate FAF and VA although confluent hypoautofluorescence had been shown to be a poor prognostic factor in chronic CSCR.

The meaning of these findings is twofold: the analyzed FAF signal derives from lipofuscin accumulating within the RPE cells. Therefore, a lower signal indicates a reduced RPE metabolism or less oxidative stress of the overlying photoreceptors. A similar theoretical model explains the nonfluorescence of geographic atrophy in age-related macular degeneration (AMD) [22]. Nevertheless, the central visual acuity in eyes compromised by advanced dry AMD is limited and the FAF signal is almost always extinct. The herein investigated eyes gained visual acuity and hence benefitted from treatment. A recent review on evidence-based medicine considers acute CSCR as self-limiting and best observed in the majority of cases [23]. Nevertheless, reduced-fluence PDT with verteporfin reflects a considerable treatment option for acute CSCR as published by our study group [24]. Positive treatment effects concerning macular sensitivity and fixation stability have already been investigated in eyes with acute CSCR 6 months after half-fluence PDT [9]. Von Rückmann et al. investigated abnormalities in different conditions of CSCR and related increased FAF to higher metabolic activity of the RPE [25]. Areas of increased FAF returned to baseline intensity 1 month after standard PDT as shown by Ozmert and his study group [17]. Normalized SW-FAF GV as a more precise predictive factor for therapeutic outcomes 1 year after half-fluence PDT for the treatment of acute CSCR were shown to be a promising diagnostic feature as published in 2015 by our study group [18]. Though these findings represented photoreceptor function and did not indicate negative treatment effects on respective parameters, no conclusions concerning a long-term effect on RPE function could be drawn.

Another comprehensible theory relies on the large time interval between the two measurements. Quantitative FAF was introduced in 2011 and performed by calibrating the FAF image to an embedded reference of known fluorescence [14]. Thus, made it possible to quantify and compare FAF intensity between patients and across short time periods. Time is an essential and relevant factor for lipofuscin deposition in the RPE and hence for the evaluation of SW-FAF GV. Long-standing SRF or sub-RPE fluid appears hyperfluorescent in FAF pictures. Matsumoto et al. observed an increased patchy FAF in the majority of eyes compromised with primary CSCR [26]. It has been hypothesized to be the result of accumulations of unphagocyted photoreceptor outer segments due to an elevation of the neurosensory retina and the disruption of the outer segment/RPE-cell junction [19]. Freund et al. postulated hyperautofluorescent signals to be a reduction in optical pigment density that affects photoreceptors prior to RPE cells [27]. Autofluorescence of macrophages from the subretinal space in SRF taps after rhegmatogenous retinal detachments has been verified in a small cohort [28]. The restoration of the RPE/retina barrier can be visualized by structural OCT despite continuous hyperfluorescence throughout a longer observation period. Therefore, its function following complete integrity might not be effectively evaluated by FAF measurements 1 year after therapy. This concept would contribute to our data published in 2015 [18]. The normalized SW-FAF GV after 83 months were reduced compared to values measured 1 month and 1 year after half-fluence PDT. Both, SW-350 and SW-1200 of the affected eye, were significantly lower in contrast to the contralateral unaffected eye, which also demonstrated degradation of SW-FAF GV over time in a nonsignificant matter. Considering the homogenous scatter plot distribution shown in Figure 2, a similar reduction of SW-FAF GV becomes evident in all eyes independent of its past history. In our opinion, most tested eyes demonstrated lower normalized SW-FAF GV meaning an FAF fovea/30° area ratio increase over time with aggravation in eyes formerly compromised by acute CSCR after treatment. It is unknown whether the amount of FAF equals those in healthy eyes of a comparable study cohort after adjusting for age [20].

Our study has several limitations. Its retrospective design questions many other factors likely to be evaluated in a prospective study. The sample size is small and does not reflect a comprehensive study population. Its analysis was not appropriate for testing of descriptive data like spot size or age and their possible influence on our results. Missing data could impact statistical analysis. No comparison for matched healthy study samples was conducted, and hence a conclusion towards a normal spectrum of FAF signal cannot be drawn. This study's strength is its long observation period and data recording by means of well-established repeatable methods and personnel. To our knowledge, this is the first study on long-term data after half-fluence PDT for acute CSCR with favorable outcomes concerning functionality in spite of SW-FAF GV differences.

## 5. Conclusions

7 years after treatment of acute CSCR with reduced-fluence PDT, normalized SW FAF-GV were significantly lower compared to 1 month after therapy. The calculated differences seem independent of the event of acute CSCR or its treatment as no explicit pattern changes in FAF or structural damages in SD-OCT could be elaborated. The contralateral untreated eye experienced a similar signal reduction after 7 years. Half-fluence PDT remains a safe and considerable option for therapeutic management of acute CSCR.

## Figures and Tables

**Table 1:** Data of patients treated with half-fluence PDT for unilateral acute CSCR.

*n*	Gender	Age	Laterality	Spot	Follow-up^a^	BCVA^b^	BCVA^c^	SW-350^d^	SW-350^c^	SW-1200^d^	SW-1200^c^
1	m	43	OD	2000	89	0.96	1.16	0.56	0.53	0.9	0.76
			OS					0.58	0.45	0.96	0.77
2	f	44	OD					0.23	0.27	0.37	0.43
			OS	2600	88	0.96	1.18	0.53	0.26	0.54	0.43
3	f	49	OD	1500	85	0.80	1.30	0.38	0.28	0.69	0.52
			OS					0.32	0.21	0.65	0.42
4	f	42	OD	1600	84	1.02	1.06	0.34	0.46	0.44	0.63
5	m	43	OD	1900	85	1.22	1.30	0.46	0.48	0.74	0.79
			OS					0.4	0.32	0.63	0.52
6	m	57	OD	1900	83	0.50	0.88	0.41	0.32	0.58	0.36
7	m	46	OD	2500	83	1.14	1.14	0.78	0.78	1.17	0.98
			OS					0.57	0.67	0.81	0.95
8	m	44	OD	1500	84	1.08	1.16	0.44	0.35	0.72	0.56
			OS					0.48	0.43	0.72	0.72
9	m	41	OD	1500	83	1.20	1.30	0.47	0.32	0.78	0.51
			OS					0.52	0.41	0.84	0.69
10	m	52	OD	2100	80	1.20	1.30	0.37	0.36	0.57	0.63
			OS					0.3	0.43	0.61	0.67
11	f	67	OD					0.69	0.34	0.69	0.61
			OS	1500	77	0.62	1.18	0.51	0.33	0.83	0.58

PDT = photodynamic therapy; CSCR = central serous chorioretinopathy; *n* = patient number; spot = spot size in *μ*m; *a* = in months; BCVA = best-corrected visual acuity in letters ETDRS; ETDRS = the Early Treatment Diabetic Retinopathy Study scale; *b* = before treatment; *c* = at follow-up; SW-350/-1200 = normalized short wavelength fundus autofluorescence grey values for 350 *μ*m and 1200 *μ*m; *d* = one month after treatment; *m* = male; *f* = female; OD = oculus dexter; OS = oculus sinister.

## Data Availability

The data used to support the findings of this study are available from the corresponding author upon request.
